# Supplementary material for: Rac Inhibition Causes Impaired GPVI Signalling in Human Platelets through GPVI Shedding and Reduction in PLCγ2 Phosphorylation
Source: Int J Mol Sci. 2022 Mar 29;23(7):3746. doi: 10.3390/ijms23073746 (PMC8998833; doi:10.3390/ijms23073746)
Supplement: Supplementary file 1 [file ijms-23-03746-s001.zip › ijms-1639804-supplementary.pdf]

International Journal of
Molecular Sciences

MDPI

Online Supplement

Rac Inhibition Causes Impaired GPVI Signalling in Human Platelets Through GPVI Shedding and Reduction of PLCγ2 Phosphorylation

Raluca A. I. Neagoe[1,2], Elizabeth E. Gardiner[3], David Stegner[2], Bernhard Nieswandt[2], Steve P. Watson[1,3] and Natalie S. Poulter[1,3*]

Supplemntary Materials and Methods

Mouse Platelet Preparation

Washed murine platelets were prepared as described previously [52]. The platelets were diluted to 5 x 10^8/mL for the Wester Blotting assay.

Rac1 Activation assay

Rac1 activity was measured using the Rac1 Activation kit (Cell Biolabs, UK) that uses agarose beads conjugated with the p21 binding domain (PBD) of PAK to pull-down active form of Rac from platelet lysate. Then, a western blot was performed and the polyvinylidene difluoride membrane was incubate with anti-Rac1 antibody (1:500) over night at 4°C. The developing and quantification of the band intensity was performed as explained in the methods section.

Light Transmission Aggregometry

The aggregation assay of freshly washed human platelets was performed using the Chrono-Log aggregometer (USA) at 37°C and stirring at 1,200 rpm constantly. Platelets were pre-incubated with different concentrations of the Rac1 inhibitor EHT1864 (0, 3, 10, 30 and 50 µM) for 5 minutes at 37°C. Then, they were stimulated with Horm collagen (Takeda, Austria) at 1 or 10 µg/mL or CRP (CAMBOL Laboratories, UK) at 1 or 5 µg/mL. The aggregation was monitored for 6 min after the addition of the GPVI agonists.

Platelet Spreading

The spreading of human washed platelet was performed on glass coverslips uncoated or coated with Horm collagen (10 µg/mL) or fibrinogen (Enzyme Research Laboratories, UK) (100 µg/mL). Platelets were pre-incubated with EHT1684 (30 µM) for 10 min at 37°C. The spreading assay, labelling the platelets with Alexa Fluor® 488-Phalloidin, and the imaging were performed following the protocol described previously [53]. Platelet segmentation performed to analyse platelet count, surface area and degree of spreading was achieved using KNIME software version 4.0.1 [54] and ilastik version 1.3.2 [55,56].

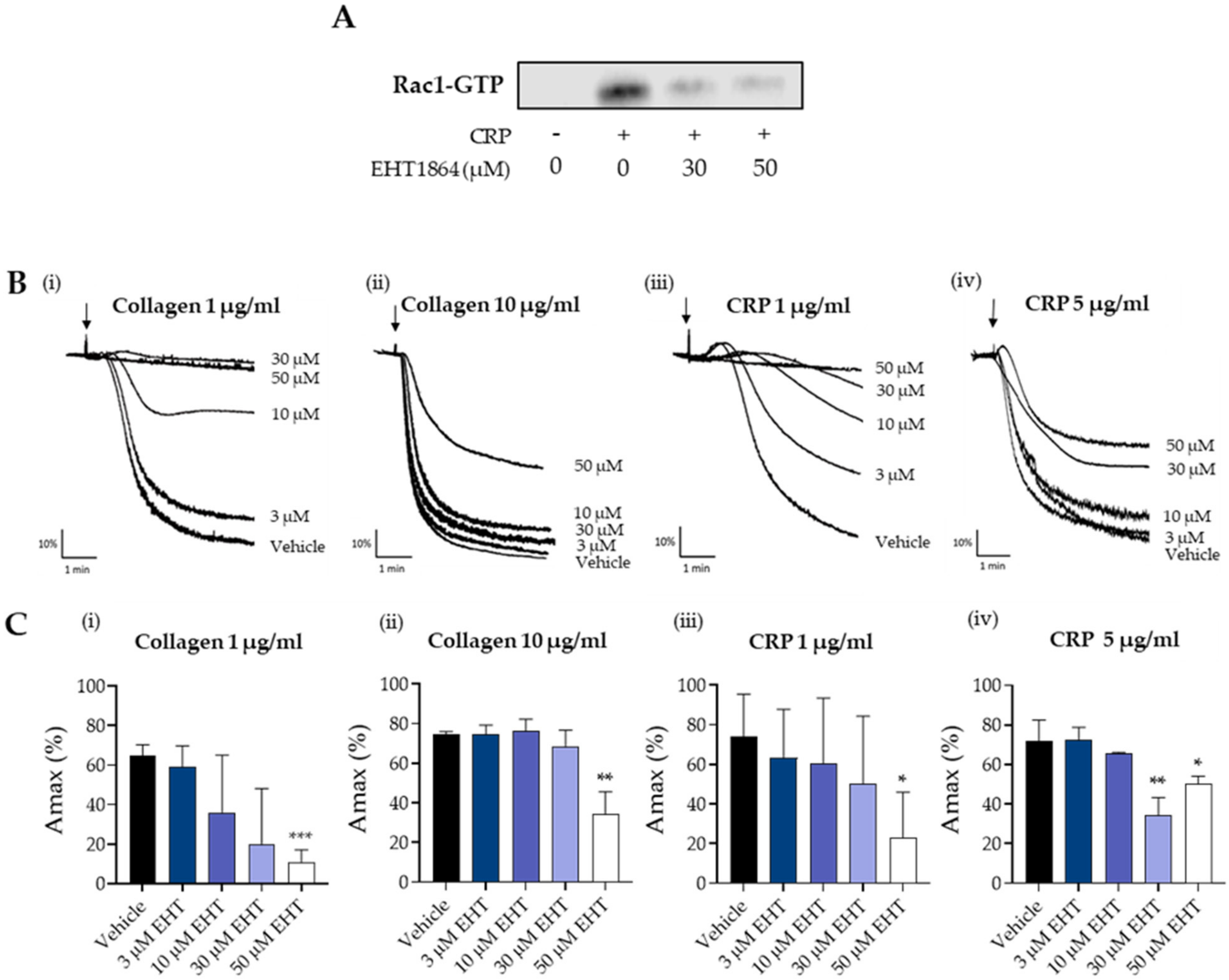

Figure S1. EHT1864 inhibits Rac1 activation and GPVI mediated aggregation on human platelets. (**A**) Dose-dependent inhibition of Rac1 activation on human platelets pre-treated with EHT1864 (30 and 50 μM) for 10 minutes and subsequently stimulated with or without CRP (5 μg/ml). Representative image of Western blotting of active Rac1-GTP. (**B**) The influence of different concentrations of EHT1864 (3, 10, 30, 50 μM) or vehicle (PBS) on Horm collagen or CRP induced platelet aggregation was recorded by light transmission aggregometry. Representative aggregation traces of stimulation with (**Ai**) Horm collagen (1 μg/ml), (**Aii**) Horm collagen (10 μg/ml), (**Aiii**) CRP (1 μg/ml) and (**Aiv**) CRP (5 μg/ml). (**Bi-Biv**) Quantification of platelet aggregation assay, showing Maximal Amplitud (Amax) of the vehicle and treated samples. (n=3), Mean ± SD * $P < 0.05$, ** $P < 0.01$, by unpaired *t*-test (comparing vehicle *vs* EHT1864 treated platelets).

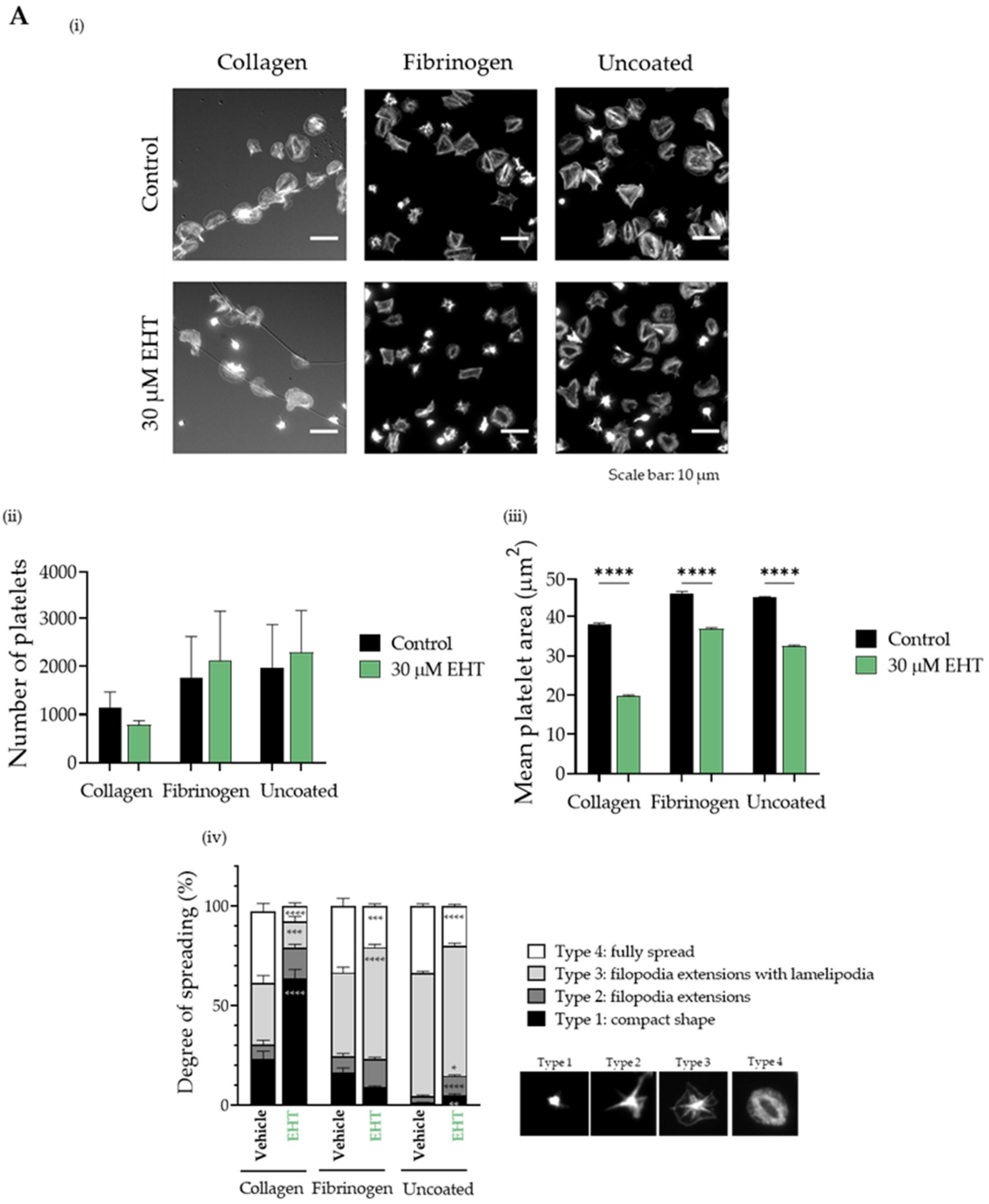

Figure S2. The effect of EHT1864 inhibiting spreading of human platelet. The effect of EHT1864 (30 µM) on the adhesion and spreading of human platelet spread on Horm collagen, fibrinogen or uncoated glass. (**Ai)** Representative panel of platelets spreading with platelets stained with Phalloidin-488. (**Aii**) Quantification of number of adherent platelets, **(Aiii)** mean platelet area and (**Aiv**) degree of spreading (compact shape, only filopodia extensions, filopodia and lamellipodia extensions and fully spread) in each treatment. Mean ± SEM * $P < 0.05$, ** $P < 0.01$, *** $P < 0.001$, **** $P < 0.0001$ by unpaired *t*-test.

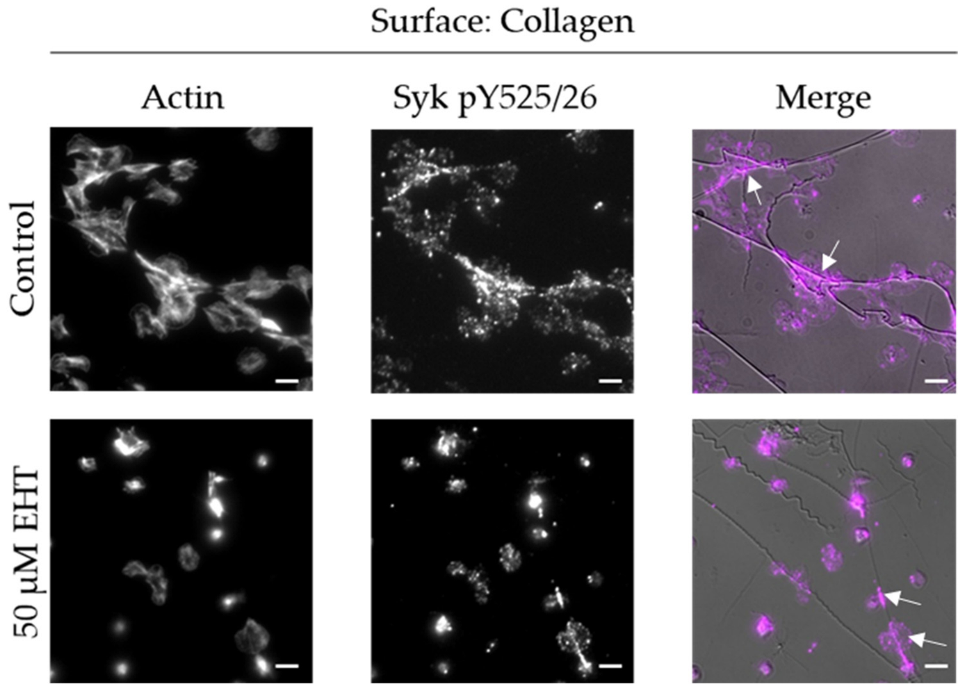

Figure S3. EHT1864 does not affect phosphorylated Syk localisation along the collagen fibres on spread human platelets. Epifluorescence microscopy imaging of human washed platelets spread on Horm collagen (10 μg/ml) pre-treated with EHT1864 (50 μM) for 10 minutes. (**A**) Representative panel of platelets spreading with platelets labelled with (**Ai)** Phalloidin-488, (**Aii**) anti-Syk pY525/26 – Alexa Fluor 647 antibody, and (**Aiii**) merge of anti-pSyk image and differential interference contract (DIC) image of the collagen fibers with arrows highlighting pSyk enrichment at collagen fibers. Scale bar: 10 μm.
